# Cauliflower Cancer

**Published:** 1894-08

**Authors:** 


CAULIFLOWER CANCER.—

℞ Liquor. potasii arsenitis....................℥j (31 grammes).
Tinct. ferri chloridi......................℥ ij (62 grammes).
Syrup simp., q. s. ad.....................℥ xx (620 grammes).

M. Sig.—Take a dessertspoonful three times a day with meals. Free use of disinfectant washes, and every three to four days the following, carefully applied with a brush:

℞ Ergotin..................................℈ iv (5.20 grammes).
Tinct. iodini comp.......................f ℥ ss (16 grammes).
Glycerini.................................f ℥ iv (124 grammes).

M. One case, after six months, well, and now, ten years later, perfectly well. Nine cases treated in the same way, and all perfectly well.—C. R. Earley, *College and Clinical Record*, March, 1894; *Univers. Med. Journal*, May, 1894.—*Ex.*

